# Intravenous Artesunate for Severe Malaria in Travelers, Europe

**DOI:** 10.3201/eid1705.101229

**Published:** 2011-05

**Authors:** Thomas Zoller, Thomas Junghanss, Annette Kapaun, Ida Gjørup, Joachim Richter, Mats Hugo-Persson, Kristine Mørch, Behruz Foroutan, Norbert Suttorp, Salih Yürek, Holger Flick

**Affiliations:** Author affiliations: Charité Universitätsmedizin, Berlin, Germany (T. Zoller, N. Suttorp, S. Yürek, H. Flick);; Universitätsklinikum Heidelberg, Heidelberg, Germany (T. Junghanss, A. Kapaun);; The State University Hospital, Copenhagen, Denmark (I. Gjørup);; Universitätsklinikum Düsseldorf, Düsseldorf, Germany (J. Richter);; Hospital of Helsingborg, Helsingborg, Sweden (M. Hugo-Persson);; Haukeland University Hospital, Bergen, Norway (K. Mørch);; Armed Forces Hospital, Berlin (B. Foroutan)

**Keywords:** artesunate, Plasmodium falciparum, parasites, malaria, hemolysis, critical care, travelers, Europe, synopsis

## Abstract

Multicenter trials in Southeast Asia have shown better survival rates among patients with severe malaria, particularly those with high parasitemia levels, treated with intravenous (IV) artesunate than among those treated with quinine. In Europe, quinine is still the primary treatment for severe malaria. We conducted a retrospective analysis for 25 travelers with severe malaria who returned from malaria-endemic regions and were treated at 7 centers in Europe. All patients survived. Treatment with IV artesunate rapidly reduced parasitemia levels. In 6 patients at 5 treatment centers, a self-limiting episode of unexplained hemolysis occurred after reduction of parasitemia levels. Five patients required a blood transfusion. Patients with posttreatment hemolysis had received higher doses of IV artesunate than patients without hemolysis. IV artesunate was an effective alternative to quinine for treatment of malaria patients in Europe. Patients should be monitored for signs of hemolysis, especially after parasitologic cure.

Infection with *Plasmodium falciparum* malaria remains a major risk for European travelers returning from malaria-endemic areas. World Health Organization (WHO) guidelines recommend intravenous (IV) artesunate as first-line therapy for severe malaria (*1*). However, quinine is still the primary treatment for severe non–multidrug-resistant *P*. *falciparum* malaria in Europe (*2*) because IV artesunate is not registered for this indication, and the only commercially available product is not manufactured according to good manufacturing practice. Quinine has several adverse effects (e.g., cardiotoxicity, hypotension, hypoglycemia, and cinchonism), has a narrow therapeutic range, and must be administered 3×/d by rate-controlled infusion (*3*,*4*). In experienced hands, adverse effects can be minimized, but a major proportion of patients still experience moderate-to-severe side effects.

The efficacy and safety of artemisinins and their derivatives in oral, rectal, and intramuscular dosage forms have been widely studied (*5*–*11*). When administered intravenously, these drugs are useful for treatment of severe malaria because of their rapid parasite clearance, apparent absence of clinically relevant side effects, and simplicity of administration (e.g., by bolus injection). Since 1992, several studies in Asia (*5*,*6*,*8*–*10*) and a recent study of children in Africa (*11*) have shown better, or at least equivalent, survival rates for patients with severe malaria treated with artesunate than for those treated with quinine. This finding applies particularly to patients with severe malaria and hyperparasitemia (*10*).

Systematic data are not available for safety and efficacy of IV artesunate for treatment of severe *P*. *falciparum* malaria outside disease-endemic areas. In the United States, use of IV artesunate is monitored by the Centers for Disease Control and Prevention (Atlanta, GA, USA) under an investigational new drug protocol (*12*). In Europe, artesunate manufactured by the Guilin Pharmaceutical Factory No. 2 (Shanghai, People’s Republic of China), which was used in all major trials of artesunate in Southeast Aia and Africa (*9*–*11*), is used. TropNetEurop (www.tropnet.net/about/contents/about_tropnet.htmla), a European surveillance network for tropical diseases, has been collecting data on artesunate use since 2005 (*13*).

Severe malaria is rare outside disease-endemic regions. Thus, the limited numbers of patients in industrialized countries makes it difficult to conduct trials with sufficient statistical power to reproduce the survival benefit for IV artesunate observed in Southeast Asia (*10*). Nonetheless, these patients may benefit from the lower cardiotoxicity of artesunate than that of quinine and, because of more rapid parasite clearance, from reduction of time spent in intensive care units, in-hospital treatment, decreased use of exchange transfusion, and secondary complications. This finding is relevant for increased numbers of older persons who travel abroad to malaria-endemic areas, despite relevant cardiac or other medical conditions associated with a several-fold increased risk for complications and death caused by severe malaria (*14*). We report data for 25 patients with severe malaria who were treated with IV artesunate in 7 treatment centers in areas to which malaria was not endemic.

## Study Characteristics

During January 2006–June 2010, we conducted a retrospective analysis of 25 patients from 7 treatment centers in Europe who were admitted to a hospital for *P*. *falciparum* malaria, which was classified as severe according to WHO criteria (*15*,*16*), and who received IV artesunate as the main antiparasitic therapy. The hyperparasitemia level for patients in a region to which malaria was not endemic was >5% (*15*). Patients treated at 7 centers, 4 in Germany (2 in Berlin, 1 in Heidelberg, and 1 in Düsseldorf), and 1 each in Denmark (Copenhagen), Sweden (Helsingborg), and Norway (Bergen), participated in the study. The Berlin (Charité University Medical Center), Heidelberg, Düsseldorf, Bergen, and Copenhagen centers are tertiary care academic teaching hospitals; the center in Helsingborg and the Armed Forces Hospital in Berlin are secondary care regional referral hospitals. The second Berlin center and the Bergen center provided data only for patients with posttreatment hemolysis; other centers provided data for all patients treated with IV artesunate. Anonymous treatment data were reported on case-reporting forms for severe malaria (TropNetEurop). The study was reviewed and approved by the ethics committee of the Charité Hospital in Berlin. Artesunate was obtained from the Guilin Pharmaceutical Factory No. 2 and stored at room temperature in all centers, according to the manufacturer’s instructions.

## Posttreatment Hemolysis

Serum and plasma of 3 patients with unusual posttreatment hemolysis in Berlin and Heidelberg (patients 6, 7, and 9) were tested for drug-induced autoantibodies, which react in the absence of the drug or its metabolites with erythrocytes, and for drug-dependent antibodies, which react only in the presence of the drug or its metabolites. Serum or plasma samples were available for testing from the time of artesunate treatment (patient 7), from the period of posttreatment hemolysis (patients 6, 7, and 9), or from the convalescent phase (7 and 16 months; patients 6 and 9).

Serologic testing was conducted by using standard gel card techniques (DiaMed, Cressier sur Morat, Switzerland). Artesunate was diluted in 0.9% NaCl at a concentration of 1.0 mg/mL. Ex vivo antigens (urine) were obtained from 2 patients receiving IV artesunate to detect reactivity to artesunate metabolites. Serum samples were tested for reactivity with artesunate solution or urine metabolites by using the indirect antiglobulin test and a drug-dependent–antibody test with the gel card technique (*17*–*20*). Cumulative doses and treatment duration (days) were compared between adult patients with and without signs of posttreatment hemolysis by using the Mann-Whitney U test.

## Patient Characteristics

One child and 24 adults (mean ± SD age 44.1 ± 16.1 years; 14 male and 11 female patients) treated with IV artesunate for severe malaria during January 2006–June 2010 were included in the study (Table A1). Eighteen patients were travelers from Europe to malaria-endemic areas, and 7 patients were immigrants who returned from malaria-endemic countries after having visited friends and relatives. With the exception of patient 13, who was a short-term visitor to Germany from Chad, all other patients who visited friends and relatives had permanently left their home countries for >5 years before becoming infected.

Hyperparasitemia (range 5%–51% parasitized erythrocytes) in 20 (80%) patients and cerebral malaria in 8 (32%) patients were the most common severe malaria-defining criteria observed. Seven patients (28%) had renal failure, and 2 (8%) required hemodialysis. Respiratory failure caused by severe shock developed in 1 patient; this patient required therapy with vasopressors and mechanical ventilation for 6 days. Repeated chest radiographs did not show pulmonary edema or pneumonia. Shock developed in 4 patients (patients 3, 4, 13, and 25); these patients required vasopressor therapy.

## Antimalarial Therapy

Details on dosage, treatment duration, and concomitant therapy are shown in Table A1. All but 3 patients received IV artesunate as first-line therapy. Therapy for patient 1 was changed to IV artesunate after complications (bradycardia) caused by the first dose of quinine. Therapy for patients 10 and 13 was 1 dose of artemether/lumefantrin or IV quinine, respectively, before transfer to a treatment center to avoid a delay in treatment initiation.

Patients 3–8, 15, 16, 18, and 19 received the dosing regimen for artesunate initially recommended by WHO (*16*): after an initial dose of 2.4 mg/kg, therapy was continued with 1.2 mg/kg every 12 hours and then 1.2 mg/kg every 24 hours. Patients 9–13 and 19–25 received artesunate, 2.4 mg/kg/dose. Therapy for all but 6 patients was changed to oral artemether/lumefantrine or atovaquone/proguanil after rapid clinical improvement and ability to swallow on days 3–4 of treatment. Different batches of artesunate were used in the Berlin and Heidelberg treatment centers. Batch information was not available from centers in Helsingborg, Copenhagen, and Bergen and the second center in Berlin. Six patients in whom posttreatment hemolysis occurred were treated for 4 years.

## Efficacy

In all patients with hyperparasitemia, parasite load was reduced ≈1 log_10_ after 24–36 hours. All but 1 patient were free of parasites 36 hours–134 hours after the initial dose of artesunate. Parasite clearance was delayed (158 hours) in 1 patient (patient 7). In this patient, infection with HIV was diagnosed (CD4 count 382 cells/µL). Mean ± SD parasite clearance time was 81.2 ± 35.4 hours for all patients treated with IV artesunate as first-line drug, who had an initial parasitemia levels >1% and for whom data were available (patients 2–12, 14, and 20–24), and 78.9 ± 29.5 hours for patients not infected with HIV.

## Tolerability

IV artesunate was generally well tolerated; there was no evidence of hemodynamic, cardiac, or allergic adverse reactions. Six patients from 5 treatment centers showed unusual hemolytic anemia, which recurred after clearance of parasites and was diagnosed 14–31 days after the first dose of IV artesunate (patients 6, 11, and 23) or persisted after the end of treatment until the end of the fourth week after the first dose of IV artesunate (patients 7, 9, and 25). Laboratory findings and typical patterns of hemolysis are shown in the Table and the Figure.

**Figure:**
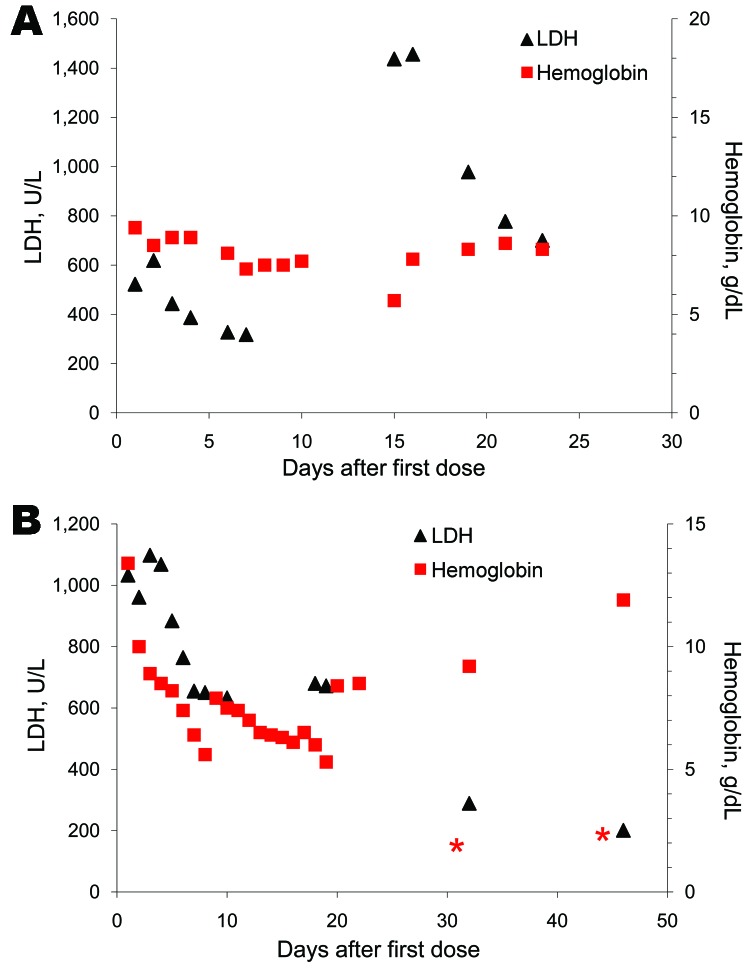
Typical patterns of hemolysis in 2 travelers with severe malaria treated with intravenous artesunate, Europe, January 2006–June 2010. A) Patient 6 with recurring hemolysis. B) Patient 9 with persisting hemolysis. LDH, lactate dehydrogenase. * indicates blood transfusion. Gaps between symbols indicate periods when samples were not obtained.

**Table:** Laboratory test results for 6 patients with posttreatment hemolysis who had been treated with intravenous artesunate for severe malaria, Europe, January 2006–June 2010*

Patient no.	Initial parasitemia level, %	Levels at first examination		Treatment duration, d	Parasite clearance, d	Levels at end of treatment		Day of diagnosis of hemolysis†	Levels at diagnosis of hemolysis		Other test results
		Hb, g/dL	LDH, U/L			Hb, g/dL	LDH, U/L		Hb, g/dL	LDH, U/L	
6	30	11.3	765	7	4	7.7	317	15	5.7	1,437	Coombs negative, reticulocytes 10.2%, G6PD deficiency ruled out
7	20	13.2	1,359	7	7	8.2	NA	32‡	6.1	805	None
9	30	13.4	1,033	4	5	7.6	650	19‡	5.3	672	None
11	4	13.4	904	7	2	9.8	311	15	7.8	660	Standard reticulocyte count
23	9	15.5	490	4	2	11.1	571	15	5.7	1,489	Reticulocytes >2× upper reference value, haptoglobin <0.1g/L, Coombs negative
25	10	14.2	570	3	NA	7.8	454	16‡	5.8	444	Reticulocytes 3× upper reference value, haptoglobin <0.08 g/L (day14), G6PD deficiency ruled out

*Hb, hemoglobin; LDH, lactate dehydrogenase; G6PD, gluose-6-phosphate dehydrogenase; NA, not available. †After first dose of artesunate. ‡Patients had persistent hemolytic activity after the end of malaria treatment.

Patient 6 was treated with artesunate and doxycycline; she had malaria-related hemolysis and an initial hemoglobin level of 11.3 g/dL. This patient was discharged in good clinical condition on day 10 (hemoglobin level 7.7 g/dL, which had been stable for the past 4 days) and had a decreased lactate dehydrogenase (LDH) level (317 U/L). On day 15, this patient was readmitted because of severe anemia caused by recurring hemolysis (hemoglobin 5.7 g/dL, LDH 1,437 U/L). Glucose-6-phosphate dehydrogenase (G6PD) deficiency and antibody-mediated hemolysis were excluded as causes (negative result for Coombs test). The reticulocyte count was high (10.2%). After receiving 2 units of packed erythrocytes, the hemoglobin level of this patient remained stable.

Patient 11, who was treated in Helsingborg, received IV artesunate for 7 days. On day 15, laboratory parameters were indicative of secondary hemolysis. The reticulocyte count was within reference limits initially and was near the upper reference value during secondary hemolysis. Patient 23, who was treated in Bergen, had a similar episode of recurring and intense hemolysis after 4 days of treatment with IV artesunate, beginning on day 15, which required readmission to the center and blood transfusion. Results of the Coombs test were repeatedly negative, G6PD deficiency was ruled out, and reticulocytes values were 2.3× the upper reference value.

Other patients showed patterns of persisting hemolysis. Patient 7 was discharged from the hospital in Berlin 14 days after the first dose of IV artesunate (treatment duration 7 days) with a hemoglobin level of 8.2 g/dL, which was stable for 10 days. On day 32, this patient was readmitted to the University Hospital in Heidelberg with a hemoglobin level of 6.1 g/dL and signs of hemolysis (LDH 805 U/L). The patient received 2 units of packed erythrocytes and was discharged 3 days later in good clinical condition. Hemolytic activity decreased over the next 10 days.

Patient 9 was treated in Heidelberg and received IV artesunate for 3 days, followed by oral artemether/lumefantrine for another 3 days, and was parasite free after 96 hours. Intense and persisting hemolysis resulted in a hemoglobin level of 5.6 g/dL on day 8 (LDH 633 U/L). This patient received 2 units of packed erythrocytes, and hemoglobin level increased to 7.9 g/dL. On day 19, hemoglobin level decreased to 5.3 g/dL because of persistent hemolysis (LDH 672 U/L); he was again given transfusions of packed erythrocytes. Hemolytic activity decreased after day 22, and LDH levels returned to reference values on day 46. Patient 25 showed a similar pattern of persistent hemolysis, which gradually decreased after day 21, after malaria therapy.

In multiple repeat thin blood films used to determine parasitemia levels, no abnormalities in erythrocyte morphology were observed. To identify causes of posttreatment hemolysis, cumulative doses of IV artesunate and treatment durations were compared among all adult patients. Patients with posttreatment hemolysis had received higher doses of IV artesunate than patients without observed hemolysis (mean ± SD cumulative dose 12.8 ± 3.3 mg/kg vs. 7.6 ± 2.9 mg/kg; p = 0.006 in all adult patients) and were treated for longer periods (mean ± SD 5.8 ± 1.6 days vs. 3.6 ± 1.7 days; p = 0.038).

## Immunohematologic Tests

Free indirect antibodies against globulin were not detected in serum or plasma from 3 patients (patients 6, 7, and 9) at the Berlin and Heidelberg treatment centers who had prolonged posttreatment hemolysis. Presence of drug-dependent antibodies in serum or plasma of patients was investigated by using as test substrates an artesunate solution and urine (artesunate metabolites) of patients receiving artesunate therapy; antibodies were not detected.

## Clinical Outcome

All patients survived and all complications related to severe malaria resolved at time of hospital discharge for all but 1 patient. Patient 2 had a more severe clinical course (respiratory and renal failure), and required further rehabilitation and physiotherapy because of critical illness (neuropathy) that developed while he received prolonged intensive care and immobilization. Unusual hemolysis in 6 patients resolved spontaneously during weeks 3–6 after the first dose of IV artesunate.

## Conclusions

Data from large multicenter trials on use of parenteral artesunate are limited to malaria-endemic regions, particularly Southeast Asia. We report data on use of parenteral artesunate for patients with severe malaria outside malaria-endemic areas, who were treated according to intensive care standards in Europe. In these patients, treatment with IV artesunate was effective and induced rapid parasite clearance. The only other report of a series of patients treated with IV artesunate for severe malaria outside malaria-endemic areas was from Norway; outcomes for 9 patients were good, and adverse reactions related to IV artesunate were not observed (*21*).

Parasitemia levels took longer to clear for participants in our study than those in a study in Thailand (*9*) (mean ± SD 81.2 ± 35.4 hours vs. 62.5 hours, 95% confidence interval 53.4–71.8 hours). In contrast with uncomplicated malaria, parasite clearance times for severe malaria are difficult to compare when different drug regimens (concomitant and sequence therapy) have been used. In addition, all patients in our study were considered nonimmune.

High parasitemia levels in severe malaria are more likely to develop in nonimmune patients; such patients are more likely to receive exchange transfusions. Physicians treating patients in our study decided not to use exchange transfusions because they have unproven benefits. Artesunate has been shown to be particularly effective in reducing mortality rates among patients with parasitemia levels >10% (*10*). Therefore, patients from Europe may benefit more from treatment with artesunate than with quinine.

Unusual episodes of hemolysis developed in 6 patients in our study. These patients had clinical signs caused by anemia during the third week of treatment with IV artesunate or had persistent signs of hemolytic activity until 6 weeks after the first dose of IV artesunate. In all cases, physicians in different treatment centers were unaware of other cases at that time, and hemolysis was not immediately considered to be induced by IV artesunate. Thus, a follow-up of patient serum samples and pharmacologic analysis of drugs used was not conducted. An additional case of Coombs-negative, posttreatment hemolysis in a European traveler during the third week after receiving IV artesunate/quinine therapy in Tanzania was reported from the Netherlands (R.M. Peerenboom, unpub. data). Despite these observations, it is not appropriate to infer incidence rates from our study regarding the incidence of posttreatment hemolysis in patients treated with IV artesunate because in 2 centers not all patients were available for inclusion. Known and possible causes of hemolytic anemia in association with malaria or antiparasitic therapy include blackwater fever, artemisinin-induced reticulocytopenia, direct hemolytic effects of the drug, and drug-induced immune hemolytic anemia.

G6PD deficiency is the basis for primaquine-, quinine- (*22*), and tafenoquine- (*23*) induced hemolytic anemia, but it was not identified in patients tested in our study. Blackwater fever, which causes acute hemolysis and hemoglobinuria in the early course of malaria treatment, was observed in the South East Asian Quinine Artesunate Trial (*10*) for quinine and artesunate (5% vs. 7%). Late onset, prolonged duration, and recurrence are not typical for blackwater fever as the cause of hemolysis in our patients. A temporary depression of reticulocytopoesis 3–7 days after the first dose of artemisinin derivatives has been reported in other studies (*5*,*24*,*25*). This phenomenon was not found for patients in our study for whom reticulocyte levels were determined.

IV artesunate is rapidly hydrolyzed to the active metabolite dihydroartemisinin. Because dihydroartemisinin has a short half-life (*26*), prolonged hemolysis after stopping treatment with IV artesunate and the recurring hemolysis in 2 patients suggest that a direct hemolytic effect of the drug is unlikely, although contaminants may have a longer half-life. Drug-induced immune hemolytic anemia, which involves production of drug-induced, irregular autoantibodies against erythrocytes, is typically associated with administration of different drugs (e.g., cephalosporins, quinine, penicillin, diclofenac, or rifampin) (*19*,*27*,*28*).

Formation of drug-dependent immunoglobulin (Ig) G or IgM leads to hemolysis and complement activation only in the presence of the causative drug; other drug-independent IgG types can cause hemolytic anemia in the absence of the drug (*29*). This second type of hemolysis occurs without complement activation and, in most cases, has a less severe clinical course. This unusual pattern of hemolysis was not observed in large clinical trials conducted with IV artesunate obtained from the same manufacturer in China (*9*,*10*) or in studies of oral artesunate. One case of a similar phenomenon was reported in Japan (*30*). However, in previous trials, patients were not routinely followed up, and cases of prolonged or recurring hemolysis might have been missed. Results of immunohematologic tests in our study did not indicate drug-induced or drug-dependent hemolysis. Because these tests had to be performed with frozen blood samples, results may have been influenced by the quality of the samples. However, the consistently negative Coombs test result for fresh blood from 2 of our patients with hemolysis suggests that hemolysis induced by autoimmune mechanisms is unlikely.

Patients with posttreatment hemolysis had received a higher cumulative dose of IV artesunate and were treated for longer periods. This observation supports the hypothesis that hemolysis might occur as a consequence of artesunate treatment in a dose-dependent manner. However, because this study did not have a prospective design and patients were not routinely followed up for signs of hemolysis in all centers, we cannot exclude undetected cases of hemolysis in our study.

The underlying cause of posttreatment hemolysis in our study of travelers is still unknown. Because IV artesunate currently produced in China is not manufactured according to standards of good manufacturing practice used in Europe, contaminants might have caused direct or antibody-mediated hemolysis in our patients. However, the manufacturer of IV artesunate recently passed the WHO drug prequalification program (*31*). Hemolysis in 5 centers in Europe over a period of 4 years suggests that contamination in a batch of IV artesunate is unlikely. Other reported artesunate-related adverse reactions, such as hypersensitivity reactions (*32*) and vestibulocochlear disturbances (*33*,*34*), were not observed in our study.

The role of IV artesunate for treatment of severe malaria in patients treated in Europe remains to be defined. However, it should be considered for patients with hyperparasitemia, patients with medical conditions limiting or prohibiting use of quinine, or patients in whom quinine-related adverse reactions are observed. Efficacy and safety profiles of IV artesunate should be prospectively evaluated, and patients should be monitored for signs of hemolysis after parasitologic cure. Reducing the cumulative dose of IV artesunate by early initiation of oral treatment might help reduce risk for posttreatment hemolysis. Improving availability of IV artesunate produced according to standards of good manufacturing practice used in Europe or the United States would constitute a major step in improving therapy for severe malaria.

## Appendix

**Table A1:** Characteristics of 25 patients treated with intravenous artesunate for severe malaria, Europe, January 2006–June 2010*

Patient no.	Age, y/ sex	Status	Travel destination	Initial parasitemia, %	Parasite clearance, h	Criteria for severe malaria	Therapy	Cumulative dose, mg/kg	IV artesunate treatment duration, d	Treatment-associated hemolysis	Other complications	Other medical conditions
1	62/F	VFR	Chad	9.5	NA†	Hyperparasitemia, severe anemia	Quinine, 1 dose IV† and artesunate‡/ doxycycline IV, oral artesunate/ doxycycline from day 4	4.8	3	No	Hypotonia, bradycardia associated with quinine	None
2	63/M	TR	Kenya	0.5	115	Cerebral malaria, respiratory failure, acute renal failure	Artesunate‡/ doxycycline IV	13.6	6	No	Shock, respiratory failure, renal failure, critical illness, neuropathy	Atrial fibrillation, peripheral arterial occlusive disease
3	46/M	TR	Cameroon	10.0	134	Hyperparasitemia, acute renal failure, cerebral malaria, anemia	Artesunate§/ doxycycline IV	8.4	6	No	Shock	Chronic hepatitis C
4	46/F	TR	India	1.0	118	Cerebral malaria	Artesunate§¶/ doxycycline IV plus artemether/ lumefantrine po from day 4	7.2	3	No	Shock	Arterial hypertension
5	63/M	TR	India	17.0	54	Hyperparasitemia, acute renal failure	Artesunate§/ doxycycline IV plus artemether/ lumefantrine po from day 4	6	3	No	Urinary tract infection, sepsis	Gastroenteritis
6	30/F	TR	India	20.0	79	Hyperparasitemia	Artesunate§/ doxycycline IV for 7 d	12	7	Yes	None	None
7	54/F	TR	Burkina Faso	20.0	158	Hyperparasitemia, cerebral malaria	Artesunate§/ doxycycline IV	12	7	Yes	None	HIV infection
8	35/M	VFR	Ghana	10.0	80	Hyperparasitemia	Artesunate§/ doxycycline IV plus artemether/lumefantrine po from day 3	3.6	2	No	None	None
9	32/M	VFR	Ghana	30.0	104	Hyperparasitemia	Artesunate 2.4 mg/kg,# atovaquone/ proguanil on day 5	12	4	Yes	None	None
10	36/F	VFR	Guinea-Bissau	9.0	94	Hyperparasitemia	Artesunate§**/ doxycycline IV	13.2	7	No	None	None
11	46/M	TR	Liberia	4.0	48	Renal failure, jaundice	Artesunate#	19.2	7	Yes	None	Diabetes mellitus
12	47/F	TR	Ghana	51.0	57	Hyperparasitemia, cerebral malaria	Artesunate, 2.4 mg/kg,# clindamycin,†† followed by artemether/ lumefantrine from day 3	9.6	2	No	None	None
13	19/M	VFR	Chad	30.0	NA‡‡	Hyperparasitemia, hyperbilirubinemia, cerebral malaria	Artesunate#‡‡/ doxycycline IV plus artemether/ lumefantrine from day 3	7.2	1	No	Shock, vasopressor treatment	None
14	58/F	TR	Gambia	6.0	60	Cerebral malaria, respiratory failure, shock, hyperparasitemia	Artesunate§/ atovaquone/ proguanil	7.2	6	No	Renal failure, shock, vasopressor treatment, acidosis	None
15	54/M	TR	Sierra Leone	6.0	36§§	Shock, acidosis, jaundice, hyperparasitemia	Artesunate§/ atovaquone/ proguanil	5.4	4	No	Sepsis	None
16	69/M	TR	Gambia	6.0	36§§	Renal failure, shock, jaundice, hyperparasitemia	Artesunate§/ mefloquine	7.2	3	No	Sepsis	Diabetes mellitus
17	64/F	TR	Sierra Leone	3.0	12§§	Cerebral malaria	Artesunate‡/ atovaquone/ proguanil	7.2	4	No	None	None
18	47/M	TR	Guinea-Bissau	5.0	36§§	Hyperparasitemia	Artesunate§/ atovaquone/ proguanil	3.6	2	No	Urinary tract infection	None
19	32/F	TR	Burundi	3.5	36§§	Hyperbilirubinemia	Artesunate§/ mefloquine	6	4	No	None	None
20	25/M	VFR	Guinea	9.0	88	Hyperparasitemia, hyperbilirubinemia	Artesunate#/ artemether/ lumefantrine	12	4	No	None	None
21	35/M	VFR	Togo	20.0	38	Hyperparasitemia	Artesunate#/ artemether/ lumefantrine	7.2	2	No	None	HIV infection
22	55/M	TR	Kenya	8.0	70	Hyperparasitemia	Artesunate#/ artemether/ lumefantrine	7.2	2	No	None	None
23	49/M	TR	Angola	9.0	35	Hyperparasitemia, renal failure, jaundice, DIC	Artesunate#/ doxycycline, followed by artemether/ lumefantrine on day 5¶¶	12	4	Yes	None	None
24	2/F	TR	Kenya	8.0	48	Hyperparasitemia	Artesunate#/ artemether/ lumefantrine	4.8	1	No	None	None
25	34/F	TR	Cameroon	10.0	NA	Hyperparasitemia, renal failure, shock	Artesunate#/ doxycycline IV plus artemether/ lumefantrine	9.6	3	Yes	Vasopressor treatment, sepsis	None

*IV, intravenous; VFR, travelers born in malaria-endemic countries visiting friends and relatives; NA, not available; TR, returning travelers of European origin; po, per os; DIC, disseminated intravascular coagulation. †Patient received IV artesunate after treatment was begun with IV quinine/doxycycline. The treatment regimen was changed because of quinine-associated hypotension and bradycardia during the first dose of quinine. ‡Artesunate, 1.2 mg/kg, all doses. §Artesunate, 2.4 mg/kg, first dose; 1.2 mg/kg, subsequent doses. ¶Patient received chloroquine/primaquine for 4 days before admission. #Artesunate, 2.4 mg/kg, all doses. **Patient received 1 dose of artemether/lumefantrine before transfer to avoid a delay in treatment initiation. ††Clindamycin, 20 mg/kg/d, divided into 4 doses (*20*). ‡‡Patient received 1 dose of IV quinine before transfer to the Berlin treatment center to avoid a delay in treatment initiation. §§Parasitemia level <1%. ¶¶One dose artemether/lumefantrine was given on day 2 before IV artesunate treatment was given because of deteriorating clinical status of the patient.
